# Supplementary material for: Causes and consequences of sex-chromosome turnovers in Diptera
Source: Evol Lett. 2026 Mar 12;10(3):253–68. doi: 10.1093/evlett/qrag003 (PMC13231274; doi:10.1093/evlett/qrag003)
Supplement: qrag003_Supplemental_Files [file qrag003_supplemental_files.zip › Supplementary_Datasets.zip!/Supplementary_Datasets/Dataset_4_Proportion_on_F_%onFhomolog.pdf]

Supplementary Dataset 4: Number and proportion of genes mapped to chromosomes homologous to element F in the various species under study. Species highlighted in green were used as representatives for phylogenetic inferences.

Family	Species	Number of genes on homologue of F	Proportion_mapped genes_on_F	F_is_fused	F_is_X_or_auto	Notes
Acroceridae	Acroceridae_Acrocera_orbiculus	952	0.1392627	yes	auto	
Anthomyiidae	Anthomyiidae_Pegoplata_infirma	71	0.007897664	No	X	
Asilidae	Asilidae_Dysmachus_trigonus	213	0.02725179	No	X	
Asilidae	Asilidae_Eutolmus_rufibarbis	199	0.02553901	No	X	
Asilidae	Asilidae_Machimus_atricapillus	1068	0.1374517	yes	X	
Asilidae	Asilidae_Machimus_rusticus	216	0.02793585	No	X	
Asilidae	Asilidae_Tolmerus_cingulatus	220	0.02835417	No	X	
Bibionidae	**Bibionidae_Bibio_marce**	203	0.03180323	No	X	
Bibionidae	Bibionidae_Dilophus_febrilis	165	0.02633259	No	X	
Bombyliidae	Bombyliidae_Anthrax_anthrax	873	0.1232007	yes	X	
Bombyliidae	Bombyliidae_Bombylius_discolor	808	0.1139473	yes	X	
Bombyliidae	Bombyliidae_Bombylius_major	818	0.1156511	yes	X	
Bombyliidae	Bombyliidae_Villa_cingulata	846	0.1202901	yes	X	
Calliphoridae	Calliphoridae_Bellardia_pandia	59	0.006632194	no	X	
Calliphoridae	Calliphoridae_Calliphora_vomitoria	58	0.006444444	no	X	
Calliphoridae	Calliphoridae_Protocalliphora_azurea	67	0.007512895	no	X	
Calliphoridae	Calliphoridae_Stomorhina_lunata	57	0.006384409	no	X	
Clusiidae	Clusiidae_Clusia_tigrina	102	0.01153455	unclear	na	missing F
Coelopidae	Coelopidae_Coelopa_pilipes	104	0.01229314	no	X	
**Conopidae**	**Conopidae_Conops_quadrifasciatus**	94	0.01208537	no	X	
Conopidae	Conopidae_Myopa_tessellatipennis	1653	0.2053416	yes	auto	
Conopidae	Conopidae_Myopa_testacea	1565	0.1946033	yes	X	
Conopidae	Conopidae_Physocephala_rufipes	NA	NA	unclear	na	missing F
Conopidae	Conopidae_Sicus_ferrugineus	100	0.01273723	unclear	X	X1X2 system
Conopidae	Conopidae_Thecophora_atra	3188	0.3893978	yes	auto	
**Culicidae**	**Culicidae_Anopheles_albimanus**	534	0.08586589	yes	X	
Culicidae	Culicidae_Anopheles_aquasalis	256	0.04031496	yes	X	
Culicidae	Culicidae_Anopheles_arabiensis	597	0.08929106	yes	X	
Culicidae	Culicidae_Anopheles_atroparvus	621	0.09311741	yes	X	
Culicidae	Culicidae_Anopheles_bellator	412	0.06260447	yes	X	
Culicidae	Culicidae_Anopheles_coluzzii	574	0.08693018	yes	X	
Culicidae	Culicidae_Anopheles_coustani	610	0.09011671	yes	X	
Culicidae	Culicidae_Anopheles_cruzii	471	0.07139609	yes	X	
Culicidae	Culicidae_Anopheles_darlingi	484	0.07900751	yes	X	
Culicidae	Culicidae_Anopheles_funestus	620	0.09057706	yes	X	
Culicidae	Culicidae_Anopheles_gambiae	619	0.09301277	yes	X	
Culicidae	Culicidae_Anopheles_longipalpis	673	0.09595096	yes	X	
Culicidae	Culicidae_Anopheles_maculipalpis	627	0.09226015	yes	X	
Culicidae	Culicidae_Anopheles_marshallii	619	0.09058978	yes	X	
Culicidae	Culicidae_Anopheles_merus	610	0.09069283	yes	X	
Culicidae	Culicidae_Anopheles_moucheti	627	0.09208401	yes	X	
Culicidae	Culicidae_Anopheles_nili	596	0.09115938	yes	X	
Culicidae	Culicidae_Anopheles_parensis	696	0.100375	yes	X	
Culicidae	Culicidae_Anopheles_rivulorum	498	0.07392014	yes	X	
Culicidae	Culicidae_Anopheles_stephensi	479	0.06895063	yes	X	
Culicidae	Culicidae_Anopheles_vaneedeni	636	0.09298246	yes	X	
Culicidae	Culicidae_Anopheles_ziemani	610	0.09015667	yes	X	
Diopsidae	Diopsidae_Teleopsis_dalmanni	4020	0.4603756	yes	auto	
**Dolichopodidae**	**Dolichopodidae_Dolichopus_griseipennis**	122	0.01727801	no	X	
Dolichopodidae	Dolichopodidae_Poecilobothrus_nobilitatuss	157	0.02189069	no	X	
**Drosophilidae**	**Drosophila_pseudoobscura_pseudoobscura_**	78	0.007027027	no	auto	
Drosophilidae	Drosophila_sulfurigaster_albostrigata	76	0.007481051	no	auto	
Drosophilidae	Drosophila_sulfurigaster_bilimbata_d	65	0.006290526	no	auto	
Drosophilidae	Drosophila_sulfurigaster_sulfurigaster_d	77	0.007598934	no	auto	
Drosophilidae	Drosophilidae_Drosophila_Lowei	78	0.007130451	no	auto	
Drosophilidae	Drosophilidae_Drosophila_albomicans	70	0.00689112	no	auto	
Drosophilidae	Drosophilidae_Drosophila_ananaese	77	0.006567164	no	auto	
Drosophilidae	Drosophilidae_Drosophila_athabasca	76	0.006766382	no	auto	
Drosophilidae	Drosophilidae_Drosophila_busckii	75	0.00748279	no	auto	
Drosophilidae	Drosophilidae_Drosophila_elegans	55	0.004372714	no	auto	
Drosophilidae	Drosophilidae_Drosophila_innubila	75	0.007386252	no	auto	
Drosophilidae	Drosophilidae_Drosophila_kepulauana	63	0.006170421	no	auto	
Drosophilidae	Drosophilidae_Drosophila_mauritiana	78	0.006110937	no	auto	
Drosophilidae	Drosophilidae_Drosophila_melanogaster	78	0.00606579	no	auto	
Drosophilidae	Drosophilidae_Drosophila_miranda	78	0.006996143	no	auto	
Drosophilidae	Drosophilidae_Drosophila_nasuta	78	0.007593458	no	auto	
Drosophilidae	Drosophilidae_Drosophila_pallidifrons	76	0.007376492	no	auto	
Drosophilidae	Drosophilidae_Drosophila_sechellia	78	0.006125815	no	auto	
Drosophilidae	Drosophilidae_Drosophila_subpulchrella	77	0.006228262	no	auto	
Drosophilidae	Drosophilidae_Drosophila_teissieri	79	0.006240619	no	auto	
Drosophilidae	Drosophilidae_Drosophila_virilis	78	0.007527504	no	auto	
Dryomyzidae	Dryomyzidae_Dryomyza_anilis	92	0.0101769912	no	X	
Empididae	Empididae_Empis_livida	46	0.006249151	no	X	Large part of F in NA?
**Empididae**	**Empididae_Rhamphomyia_laevipes**	119	0.01619488	no	X	
Heleomyzidae	Heleomyzidae_Suillia_variegata	81	0.009328573	no	X	
Hippoboscidae	Hippoboscidae_Ornithomya_chloropus	47	0.00598345	no	auto	Dot-like_reversal
Hippoboscidae	Hippoboscidae_Ornithomya_fringillina	47	0.006004855	no	auto	Dot-like_reversal
Hybotidae	Hybotidae_Hybos_culiciformis	105	0.01451479	no	X	
**Lauxaniidae**	**Lauxaniidae_Calliopum_simillimum**	93	0.01034828	no	X	

Lauxaniidae	Lauxaniidae_Meiosimyza_decipiens	108	0.01204013	no	X	
Muscidae	Muscidae_Polietes_domitor	1622	0.1804628	yes	auto	
Pallopteridae	Pallopteridae_Toxonevra_muliebris	82	0.009031832	no	X	
Pipunculidae	Pipunculidae_Nephrocerus_scutellatus	163	0.0193863	no	X	
**Polleniidae**	**Polleniidae_Pollenia_amentaria**	61	0.006813359	no	X	
Polleniidae	Polleniidae_Pollenia_angustigena	64	0.007119813	no	X	
**Ptychopteridae**	**Ptychopteridae_Ptychoptera_albimana**	208	0.03193613	no	X	
Ptychopteridae	Ptychopteridae_Ptychoptera_contaminata	224	0.033929112	no	X	
Rhagionidae	Rhagionidae_Rhagio_lineola	241	0.03153212	no	X	
**Rhinophoridae**	**Rhinophoridae_Melanophora_roralis**	65	0.007418398	no	X	
Rhinophoridae	Rhinophoridae_Phyto_melanocephala	72	0.008139272	no	X	
**Sarcophagidae**	**Sarcophagidae_Sarcophaga_caerulescens**	65	0.00736377	no	X	
Sarcophagidae	Sarcophagidae_Sarcophaga_roselle	71	0.008032583	no	X	
Sarcophagidae	Sarcophagidae_Sarcophaga_subvicina	67	0.007635328	no	X	
Sarcophagidae	Sarcophagidae_Sarcophaga_variegata	37	0.0042012036	no	X	large part of F in NA?
**Sciaridae**	**Sciaridae_Bradysia_coprophila**	1739	0.2440702	yes	auto	
Sciaridae	Sciaridae_Pseudolycoriella_hygida	1487	0.2152888	yes	auto	
Sciomyzidae	Sciomyzidae_Coremacera_marginata	112	0.01249164	no	X	
Sciomyzidae	**Sciomyzidae_Pherbina_coryleti**	114	0.01292224	no	X	
Sciomyzidae	Sciomyzidae_Tetanocera_ferruginea	108	0.01207243	no	X	
**Stratiomyidae**	**Stratiomyidae_Beris_chalybata**	204	0.02640435	no	X	
Stratiomyidae	Stratiomyidae_Beris_morrisii	200	0.02560492	no	X	
Stratiomyidae	stratiomydae_Chorisops_tibialis	190	0.02430289	no	X	
Stratiomyidae	Stratiomyidae_Nemotelus_nigrinus	195	0.025886101	no	X	
Stratiomyidae	Stratiomyidae_Odontomyia_ornata	198	0.02579805	no	X	
**Syrphidae**	**Syrphidae_Blera_fallax**	157	0.01868825	no	X	
Syrphidae	Syrphidae_Brachypalpus_laphriformis	163	0.01910679	no	X	
Syrphidae	Syrphidae_Cheilosia_impressa	164	0.01953544	no	X	
Syrphidae	Syrphidae_Cheilosia_pagana	142	0.01691483	no	X	
Syrphidae	Syrphidae_Cheilosia_soror	160	0.01912503	no	X	
Syrphidae	Syrphidae_Cheilosia_urbana	156	0.01858028	no	X	
Syrphidae	Syrphidae_Cheilosia_vulpina	162	0.01940585	no	X	
Syrphidae	Syrphidae_Chrysotoxum_bicinctum	152	0.01839748	no	X	
Syrphidae	Syrphidae_Criorhina_berberina	162	0.01931791	no	X	
Syrphidae	Syrphidae_Dasysyrphus_albostriatus	149	0.01798214	no	X	
Syrphidae	Syrphidae_Epistrophe_grossulariae	147	0.01785281	no	X	
Syrphidae	Syrphidae_Epistrophella_euchroma	152	0.01841308	no	X	
Syrphidae	Syrphidae_Episyrphus_balteatus	150	0.01821272	no	X	
Syrphidae	Syrphidae_Eristalinus_sepulchralis	145	0.01749095	no	X	
Syrphidae	Syrphidae_Eristalis_arbustorum	163	0.01948595	no	X	
Syrphidae	Syrphidae_Eristalis_pertinax	164	0.01971628	no	X	
Syrphidae	Syrphidae_Eristalis_tenax	154	0.01848961	no	X	
Syrphidae	Syrphidae_Eupeodes_corollae	144	0.01750122	no	X	
Syrphidae	Syrphidae_Eupeodes_latifasciatus	164	0.019914997	no	X	
Syrphidae	Syrphidae_Leucozona_laternaria	157	0.0189362	no	X	
Syrphidae	Syrphidae_Melanostoma_mellinum	161	0.01920554	no	X	
Syrphidae	Syrphidae_Meliscaeva_auricollis	147	0.0179312	no	X	
Syrphidae	Syrphidae_Myathropa_florea	156	0.01867816	no	X	
Syrphidae	Syrphidae_Neoascia_interrupta	146	0.0173191	no	X	
Syrphidae	Syrphidae_Platycheirus_albimanus	161	0.01952462	no	X	
Syrphidae	Syrphidae_Rhingia_campestris	171	0.020284698	no	X	
Syrphidae	Syrphidae_Scaeva_pyrastri	146	0.01804251	no	X	
Syrphidae	Syrphidae_Sphaerophoria_taeniata	147	0.01781386	no	X	
Syrphidae	Syrphidae_Syritta_pipiens	158	0.01884992	no	X	
Syrphidae	Syrphidae_Volucella_inanis	174	0.02078356	no	X	
Syrphidae	Syrphidae_Volucella_inflata	168	0.02013182	no	X	
Syrphidae	Syrphidae_Xanthogramma_pedissequum	146	0.01785933	no	X	
Syrphidae	Syrphidae_Xylota_sylvarum	159	0.018879126	no	X	
**Tachinidae**	**Tachinidae_Cistogaster_globosa**	70	0.008032129	no	X	
Tachinidae	Tachinidae_Cochliomyia_hominivorax	64	0.007275207	no	X	
Tachinidae	Tachinidae_Epicampocera_succincta	68	0.008000941	no	X	
Tachinidae	Tachinidae_Gymnosoma_rotundatum	68	0.00777854	no	X	
Tachinidae	Tachinidae_Lypha_dubia	70	0.008052456	no	X	
Tachinidae	Tachinidae_Nowickia_ferox	59	0.006768384	no	X	
Tachinidae	Tachinidae_Tachina_fera	65	0.00744218	no	X	
Tachinidae	Tachinidae_Tachina_lurida	59	0.006745941	no	X	
Tachinidae	Tachinidae_Thecocarcelia_acutangulata	71	0.008283748	no	X	
Tachinidae	Tachinidae_Thelaira_solivaga	81	0.009460406	no	X	
Tephritidae	Tephritidae_Anastrepha_ludens	90	0.01011236	no	X	
Therevidae	Therevidae_Thereva_nobilitata	207	0.02669934	no	X	
**Tipulidae**	**Tipulidae_Nephrotoma_appendiculata**	121	0.01693018	no	X	
Tipulidae	Tipulidae_Nephrotoma_flavescens	120	0.01679496	no	X	
